# Predictive models for pressure ulcers from intensive care unit electronic health records using Bayesian networks

**DOI:** 10.1186/s12911-017-0471-z

**Published:** 2017-07-05

**Authors:** Pacharmon Kaewprag, Cheryl Newton, Brenda Vermillion, Sookyung Hyun, Kun Huang, Raghu Machiraju

**Affiliations:** 1 Department of Computer Science and Engineering, The Ohio State University, Columbus, Ohio USA; 2 Department of Critical Care Nursing, The Ohio State University Wexner Medical Center, Columbus, Ohio USA; 3 College of Nursing, The Ohio State University, Columbus, Ohio USA; 4 Department of Health Services Nursing Education, The Ohio State University Wexner Medical Center, Columbus, Ohio USA; 5 College of Nursing, Pusan National University, Busan, South Korea; 6 Department of Biomedical Informatics, The Ohio State University, Columbus, Ohio USA

**Keywords:** Pressure ulcers, Intensive care units, Electronic health records, Bayesian networks, Model learning

## Abstract

**Background:**

We develop predictive models enabling clinicians to better understand and explore patient clinical data along with risk factors for pressure ulcers in intensive care unit patients from electronic health record data. Identifying accurate risk factors of pressure ulcers is essential to determining appropriate prevention strategies; in this work we examine medication, diagnosis, and traditional Braden pressure ulcer assessment scale measurements as patient features. In order to predict pressure ulcer incidence and better understand the structure of related risk factors, we construct Bayesian networks from patient features. Bayesian network nodes (features) and edges (conditional dependencies) are simplified with statistical network techniques. Upon reviewing a network visualization of our model, our clinician collaborators were able to identify strong relationships between risk factors widely recognized as associated with pressure ulcers.

**Methods:**

We present a three-stage framework for predictive analysis of patient clinical data: 1) Developing electronic health record feature extraction functions with assistance of clinicians, 2) simplifying features, and 3) building Bayesian network predictive models. We evaluate all combinations of Bayesian network models from different search algorithms, scoring functions, prior structure initializations, and sets of features.

**Results:**

From the EHRs of 7,717 ICU patients, we construct Bayesian network predictive models from 86 medication, diagnosis, and Braden scale features. Our model not only identifies known and suspected high PU risk factors, but also substantially increases sensitivity of the prediction - nearly three times higher comparing to logistical regression models - without sacrificing the overall accuracy. We visualize a representative model with which our clinician collaborators identify strong relationships between risk factors widely recognized as associated with pressure ulcers.

**Conclusions:**

Given the strong adverse effect of pressure ulcers on patients and the high cost for treating pressure ulcers, our Bayesian network based model provides a novel framework for significantly improving the sensitivity of the prediction model. Thus, when the model is deployed in a clinical setting, the caregivers can suitably respond to conditions likely associated with pressure ulcer incidence.

## Background

### Pressure ulcers

A pressure ulcer (PU) is a localized injury to the skin and/or underlying tissue usually over a bony prominence, as a result of pressure, possibly in combination with shear [1]. Surveys indicate that patients admitted to the intensive care unit (ICU) have higher incidence of PUs than general hospital patients in acute care settings [2]. The prevalence of ICU pressure ulcers in the United States during the year 2009 ranged from 16.6% to 20.7% [2]. An estimated 2.5 million patients are treated each year in acute care settings at a cost of $11 billion per year due to PUs [3], many of which may be preventable.

Identifying accurate risk factors of PUs is essential to determining appropriate prevention strategies. Risk factors for ICU patients are likely to be different from those of general hospital patients since ICU patients are often in more morbid conditions and are hence more likely to be advised for bed rest. Consequently, the supine and sedentary position required of a bed rest in conjunction with related factors often leads to the onset or aggravation of PUs. The Braden scale is a risk assessment tool that can assist nurses in identifying a patient’s risk of developing a pressure ulcer [4]. It is the most widely used tool for predicting PU risk in the United States, and contains 6 predominantly skin-related criteria, namely, sensory perception, moisture, activity, mobility, nutrition, and friction & shear. Despite its widespread use, our studies found that while Braden scale is sensitive, its accuracy is considered insufficient (0.672 AUC) for identifying patients at risk for developing PUs in ICU settings [5, 6].

Besides the characteristics used in Braden scale, medications such as vasopressor agents are often administered in ICU to increase blood pressure for patients in hypotension caused from shock states. Some studies have found that vasopressor agents were statistically significant for the development of PUs in ICU settings [7, 8]. However, other studies did not find a conclusive relationship between vasopressor agents and PU development [9–11]. Separately, comorbid conditions such as hypertension, spinal cord injury, respiratory disease, vascular disease, and diabetes mellitus have also been found to increase risk of PU development [12–15]. Thus, there is a need for identifying medications and diagnoses as factors for PUs. This issue motivates us to also examine medications and diagnoses as indicators of PU incidence.

### Bayesian network

A practical problem with the use of medication and diagnosis data is that they are embedded in electronic health records (EHRs). EHRs consist of rich and comprehensive patient-specific information from a large number of sources in different formats consisting of heterogeneous data types. Even when expertly extracted out into features, many of the features (e.g. flu and high body temperature) violate the assumption of independence required by most machine learning models. Bayesian network models, on the other hand, take into account these interactions between variables and eliminate interactions between variables that fail a dependence test, while retaining more important ones.

A Bayesian network is naturally suited to represent dependent relationships between variables. Nodes in a Bayesian network represent features from the data, and edges represent dependencies between those features. Bayesian networks are interpretable, which is critical for clinicians who need a parsimonious view of probable causal factors pertaining to diseases. Bayesian networks have been applied to effectively assist users in identifying faulty network structures and model discrepancies [16], and to depict the underlying uncertainty and facilitate contextual understanding in clinical practice guidelines [17]. They have also been utilized for investigating complex phenotype data, specifically for the visualization of complex associations [18], the dependency structure of data, the reduction of dimensionality and comparison of substructures, and the estimation of causal effects from data [19]. In addition, a Bayesian network has been used to predict mortality, readmission, and length of stay in real time using EHR data to improve quality of care in the emergency unit [20].

In this paper we construct Bayesian networks from features of ICU patients in order to predict PU incidence and better understand the structure of related features. Specifically, we estimate the value of a hidden node (PU), given the values of the observed nodes (Braden scale, medication, and diagnosis features). After constructing the Bayesian network, we perform inference to estimate the risk that patients will develop PUs during hospitalization. We find that on our ICU data, Bayesian network models have comparable overall performance to, but higher sensitivity than, models constructed from classical machine learning algorithms such as logistic regression [6]. A beneficial “byproduct” of Bayesian network modeling is that a feature dependency graph structure is learned. From this feature relationship graph, high risk factors associated with PU incidence in ICU settings can be directly identified using the Markov blanket property of the Bayesian network’s PU node [21].

The Markov blanket of a node in a Bayesian network corresponds to a set of features that have high predictive power together. Specifically, the Markov blanket of a node is the set of nodes that shield the node from the rest of the network. Thus, nodes in a Markov blanket will likely predict the behavior of the node of interest. This property is very useful to identify a succinct set of features or variables highly associated with the feature-of-interest. The Markov blanket has been utilized in identifying the most critical genes towards the development of astrocytic tumors from a significant set of meta-analysis genes [22] and single nucleotide polymorphism (SNP) biomarkers significantly associated with Alzheimer’s disease [23]. Markov blanket property along with the Tabu search algorithm has been used to predict postoperative morbidity of heart disease [24].

Our contributions in this paper are the following. We evaluate our three-stage framework for predictive analysis with Bayesian networks, on one of the largest datasets developed for PU predictive analysis, which includes 86 medication, diagnosis, and Braden features extracted from the EHRs of 7,717 ICU patients. We evaluate multiple runs of all combinations of four search algorithms, two prior structure initializations, two scoring functions, and five sets of features, and find that the best combination gives Bayesian networks with an average AUC of 0.827, which is comparable to the best classical machine learning models from our previous study [6]. We visualize a representative Bayesian network and use the Markov blanket property to identify several known and several suspected high risk factors associated with PU incidence. Our clinician collaborators used this information to identify strong relationships between risk factors widely recognized as associated with pressure ulcers.

For our data the Bayesian network based approach yields models with much higher sensitivity than the classical machine learning models (average sensitivity increased to 0.455 from 0.160). Meanwhile, there is only a slight sacrifice in specificity (average specificity is 0.908 versus 0.990) and no significant difference in overall accuracy (measured in AUC). Given the strong adverse impact of PU on patients and high cost for treating PU compared to taking preventive measures, high sensitivity of the model is preferred, because patients likely to contract PUs are likely to be predicted positive.

In summary, we demonstrate that Bayesian network method is a powerful tool in inferring predictive models for syndromes such as PU from complex clinical data. Not only can it lead to high sensitivity models, but it also enables development of new hypotheses on potential risk factors.

## Methods

### Dataset

The settings of this study are three adult ICUs at The Ohio State University Wexner Medical Center (OSUWMC). OSUWMC serves as a major referral center for patients from the entire state of Ohio and throughout the Midwest. The ICUs include 83 beds in total, admitting approximately 3,800 patients annually.

**Fig. 1:**
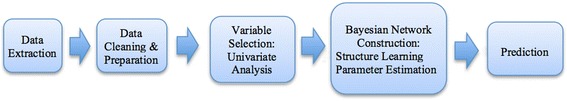
The workflow employed in this study includes modules to conduct data acquisition, data preparation, variable selection, construction of a Bayesian network, and model prediction

Essentris^©^ is the commercial system used for documentation in all ICUs at the medical center. Patient data entered into Essentris^©^ are eventually transferred to the information warehouse (IW). The IW compiles EHR data from the various electronic records systems throughout OSUWMC, namely, an administrative system (ADT^©^), a laboratory system, a computerized provider order entry (CPOE) system, and a billing system. Figure 1 shows the study procedure. Specific details will be described in the following sections.

### Data extraction

Patients (age ≥ 18) admitted to ICUs between January 1, 2007 and December 31, 2010 comprise the sample set. Institutional Review Board (IRB) approval was obtained in 2010 for the data extraction. Data were de-identified and supplied by IW staff. Patients developing a PU are identified by reviewing discharge diagnoses appropriately coded with the International Classification of Diseases (ICD)-9 codes [25] as one of the fields in EHR system. For instance, if a patient had an ICD-9 code, 707.07 (Pressure ulcer, Heel), the patient is included in the PU group. On the other hand, if a patient does not have any of the ICD-9 codes indicating PUs, the patient is included in the non-PU group.

### Data cleaning and preparation

Data cleaning and preparation processes include several steps. First, patients who are afflicted with a PU at the time of admission are excluded. In addition, patients whose ICU stay is shorter than 36 hours are excluded since PUs generally develop after 3 days of admission [26]. Second, if a patient had multiple hospitalizations during the study period, only the first hospitalization record is included. Similarly, if a patient has more than one ICU admission record during the hospitalization, only the first ICU admission record is included for analysis. This is because our objective is to find risk factors of patients who have the first incidence of PUs during their ICU stay. Patients who suffer from the onset of PUs at the time of admission are excluded as they may have previously been exposed to unknown risk factors for which we have no data. This patient selection process is consistent with practices in our previous studies [5, 6].

Medications administered to patients in PU and non-PU groups during the ICU stay are also listed. The list is reviewed by an inter-disciplinary team that includes a registered nurse, two ICU clinical nurse specialists, and a dietician. Through this manual review, medications are grouped into 72 categories based on their perceived functional purposes and efficacies. For instance, Meperidine and Nalbuphine are included in the Analgesia category, and Dopamine and Epinephrine are grouped into Vasoactive category. Medication categories are coded as dichotomous (binary) variables.

Diagnostic data in the form of ICD-9 codes are 5 digits long and are extracted from the EHR system from records within ICU length of stay. The first three digits indicate a main disease type and the last two provide additional information about the disease. Discharge diagnostic ICD-9 codes are used to identify patients with maladies during ICU hospitalization. Subsequently, ICD-9 codes are truncated into 3 digits in order to analyze the primary conditions. Most of the 707 ICD-9 codes are considered PUs except for 707.1 (ulcer of lower limb), 707.8 (chronic ulcer of other specified sites), and 707.9 (chronic ulcer of unspecified site). Those codes are labeled as 707-nonPU.

Braden scale contains 6 subscales that measure sensory perception, moisture, activity, mobility, nutrition, and friction & shear. A Braden total score is simply calculated by adding up all the subscales. We consider each subscale separately to see which subscales are more related to PU incidence in ICU settings. Moreover, most of the subscales have significant association with PU incidence, in addition to the summed Braden scale (Braden total subscale). We include these for consistency with previous work [27, 28].

### Variable selection

In order to eliminate ill-defined, non-salient, and “noise” variables irrelevant to PUs, we select a set of medications and diagnoses highly associated with the PU condition. To achieve this aim, univariate analysis is first carried out to determine what medication categories and diagnoses (variables) are highly associated with PUs. For each variable, one of the following two statistical tests is used. A χ^2^ -test, being sensitive to small expected frequencies, is used only where expected frequencies are large enough (> = 20), otherwise Fisher’s Exact Test (FET) is applied. In the midst of this screening process, we do not apply multiple test compensation in order to be more inclusive.

Medication categories that appear to be significantly associated with PUs are retained. Likewise, diagnoses that appear to be highly associated with PUs (which we call having a strong “comorbidity association”) are retained. The retained medication categories, retained diagnoses, and all Braden features are used as variables (nodes) for Bayesian networks.

### Bayesian network modeling

A Bayesian network model is introduced to model clinical data which is high dimensional in nature and characterized by variables of heterogeneous data types. Bayesian network models are graphs in which nodes represent random variables, and the lack of edges represent conditional independence. Formally, Bayesian networks are defined as follows:

Let *U* = {*x*_1_, …, *x*_*n*_}, *n* ≥ 1 be a set of random variables. A Bayesian network *B* over *U* is a network structure *B*_*s*_ in the form of a directed acyclic graph (DAG) over *U* and a set of probability assertions *B*_*p*_ = {*Pr*(*u*|*Pa*(*u*)), *u* ∈ *U*} where *Pa*(*u*) is a set of parents of *u* in *B*_*s*_.

In this work, discrete-valued Bayesian networks are used. Therefore, probability models are represented with discrete conditional probability tables. There are two steps to constructing a Bayesian network: structure learning and parameter estimation. Structure learning extracts a Bayesian network *B*_*s*_ from observed data. Parameter estimation constructs the conditional probability distribution set *U* for each node in the network once the structure has been learned.

### Structure learning

Score-based structure learning is a commonly used method to identify a network structure. This approach uses a scoring function that measures how well the model fits the observed data. The score-based structure learning assigns a score to each candidate network and tries to find the network maximizing the score. An optimal solution is intractable since this problem has been shown to be NP-Hard; therefore, many approximate methods have been proposed. Greedy hill climbing is one of the simplest and most commonly used search algorithms. It has been observed to achieve similar results as an optimal algorithm (run on small networks of not more than 20 nodes) [29]. Greedy hill climbing iteratively takes the step that leads to the largest improvement in the score until no modification improves the score. It therefore can terminate in a local optimum. Repeated hill climbing can be used to avoid being caught in local optima. It repeatedly uses greedy hill climbing algorithm and returns the best structure of the multiple runs. Tabu search and simulated annealing are two approaches that are commonly used to explore the region around, and therefore escape, local optima. Tabu search [30] is a variation of greedy hill climbing which keeps a list of length *L* of recently used operations such as edge addition, deletion, and reverse. For each step, it does not consider operations in the list, forcing it to explore new directions in the search space. Simulated annealing is a different hill climbing variation which starts with an initially large “temperature” parameter. When the temperature is large, the algorithm may take steps which decrease the score. As the algorithm proceeds the temperature is gradually reduced, and the search increasingly focuses only on moves that improve the score.

### Scoring functions

A scoring function is used with a search algorithm to approximate the probability of each candidate structure given the data *D*. The goal is to find a highest scoring structure *B*_s_^*^, that is:

$$ {B}_s^{*}=\underset{B_s}{ \arg\ \max } Score\left({B}_s\Big| D\right) $$

### Bayesian scoring function

The premise of the Bayesian scoring function is to compute the posterior distribution of a network from given data. The best network is the one that maximizes the posterior probability. A widely used Bayesian scoring function is the Bayesian Dirichlet with score equivalence and uniform priors (BDeu) proposed by Buntine [31]. BDeu assigns the same score to equivalent network structures and has a uniform prior distribution assumption. Therefore, BDeu has only one necessary hyper-parameter called *equivalent sample size*. The BDeu scoring function is defined as follows:

$$ BDeu\left({B}_s\Big| D\right)= \log \left( Pr\left({B}_s\right)\right)+{\displaystyle \sum_i^n}{\displaystyle \sum_j^{q_i}}\left( \log \left(\frac{\Gamma \left(\frac{N^{\hbox{'}}}{q_i}\right)}{\Gamma \left({N}_{i j}+\frac{N^{\hbox{'}}}{q_i}\right)}\right)+{\displaystyle \sum_k^{r_i}}\left( \log \left(\frac{\Gamma \left({N}_{i j k}+\frac{N^{\hbox{'}}}{r_i{q}_i}\right)}{\Gamma \left(\frac{N^{\hbox{'}}}{r_i{q}_i}\right)}\right)\right)\right) $$

where *Γ*(.) is the gamma function, *n* is the total number of variables, *r*_*i*_ is the number of possible values of variable *x*_*i*_ (e.g., 2 for a binary variable), and *q*_*i*_ is the number of possible values of *Pa*(*x*_*i*_). *N*_*ijk*_ is the number of records in the data set *D* having variable *x*_*i*_ in state *k* for which *Pa*(*x*_*i*_) has its *j* -th value. *N*_*ij*_ is calculated by summing over all states of a variable *x*_*i*_: ${N}_{i j}={\displaystyle \sum_{k=1}^{r_i}}{N}_{i j k}$. *N* ^'^ is the user-specified equivalent sample size, which expresses how much prior knowledge should be taken into account in the network structure.

### Information-theoretic scoring function

The premise of this scoring function is a tradeoff between how well the network structure fits the data and how complex the network is. This can be viewed as a log-likelihood (LL) function along with a penalty factor to address the over-fitting problem. The log-likelihood function is the log probability of *D* given *B*_*s*_ and can be calculated as:

$$ L L\left( D\Big|{B}_s\right)= \log \left( Pr\left( D\Big|{B}_s\right)\right) $$

There are several well-known information-theoretic scoring functions. In this study, we consider minimum description length (MDL) (equivalent to Bayesian information criterion (BIC) for Bayesian networks [29]) as it has been shown that it can outperform Akaike’s information criterion (AIC), Bayesian Dirichlet equivalence score (BDeu), and factorized normalized maximum likelihood (fNML) [29]. MDL scoring function is defined as follows:

$$ M D L\left({B}_s, D\right)= L L\left( D\Big|{B}_s\right)-\frac{logN}{2}\left|{B}_s\right| $$

where |*B*_*s*_| is the number of independent parameters in network *B*_*s*_. The penalty factor can be viewed as the number of bits required to encode the model.

### Parameter estimation

The conditional probability table for each variable in the network is created once the structure learning has been carried out. Direct estimates of the conditional probabilities are calculated for each node in the network structure as follows:

$$ \Pr \left( x= k\Big| Pa(x)= j\right)=\frac{N_{ij k} + N{\hbox{'}}_{ij k}}{N_{ij}+ N{\hbox{'}}_{ij}} $$

where *N* ' _*ijk*_ is a parameter used for estimating the probability tables and can be interpreted as the initial count on each value. When *N* ' _*ijk*_ = 1, *N* ' _*ij*_ = *r*_*i*_, assigning (instead of 0) a small prior to values unobserved in training data. With *N* ' _*ijk*_ = 0, maximum likelihood estimates are obtained.

### Markov Blanket

An important concept underlying a Bayesian network is that of a Markov blanket of a node. The Markov blanket of a node is a set of nodes that shield the node from the rest of the network. This set contains the node’s parents, the node’s children, and all other parents of its children. Formally, let *N* be the set of all nodes in a network and *M* be a set of nodes not containing node *x. M* is a Markov Blanket for *x* if *x* is conditionally independent of all variables in the set *N* – *M* – *x* and it is further required that *M* is minimal. This implies that a variable in a Bayesian network is conditionally independent of other variables not included in its Markov blanket. On the other hand, when the Markov blanket of a certain variable *x* is known, adding knowledge of other variables outside the Markov blanket leaves the probability of *x* unchanged [32]. This property is noteworthy since only variables in the Markov blanket are required to predict the behavior of the outcome variable. From this property, we can reduce the size of the model significantly.

## Results

### Patient demographics

A total of 7,717 ICU patients are included in the analysis. The number of patients in PU group is 590, while the number of patients in non-PU group is 7,127. Patient demographics are summarized in Table 1. In the patient cohort, 57.4% are male and 82.2% are ethnically classified as White. The mean age of the patients is 57.7 years and the mean length of ICU stay is 10.1 days.

**Table 1:** Demographics of ICU patients (*N* = 7717)

Variable		Total	PU group (*N* = 590)	Non-PU group (*N* = 7127)	Statistic	*p* value
Gender, freq (%)	Male	4426	378 (64.1%)	4048 (56.8%)	${x}^2=1.9$	<.001
	Female	3291	212 (35.9%)	3079 (43.2%)		
Race/Ethnicity, freq (%)	White	6345	469 (79.5%)	5876 (82.4%)	${x}^2=3.15$	.076
	Non-white	1372	121 (20.5%)	1251 (17.6%)		
Age (years), mean (SD)		57.7 (15.9)	59.0 (15.5)	57.6 (16)	t = 4.52	.034
Length of ICU stay (days), mean (SD)		10.1 (10)	13.4 (14.3)	9.8 (9.6)	t = 70.56	<.001

Note: SD = Standard Deviation

Table 1 shows that gender and length of ICU stay are statistically significant factors influencing PU development. However, clinicians are already attuned to the relationship between length of ICU stay or hospitalization and PU incidence. Consequently, we are looking for non-obvious relationships that could be related to PU occurrence such as medications and diagnoses.

### Medication variable selection

Medications that are used for patients in PU and non-PU groups during the ICU stay are listed. In total, 828 unique medications are administered to the patients in our study. Our research team including a registered nurse, two ICU clinical nurse specialists, and a dietician, reviewed all of the 828 medications. Through the afore-mentioned manual review, medications are grouped into 72 categories. From the list of medications, categories whose frequency is less than 10 are removed as they are not considered significant for the univariate analysis. Additionally, “Electrolytes”, “IV Fluid”, “Research Drugs”, and “Miscellaneous” categories are removed as they do not appear to be clinically meaningful. As a result, 49 categories are retained and used for univariate analysis. In general, the association of individual medication category with PU is not strong; only 18 medication categories are found to be significantly associated with PUs at a significance level of 90% (i.e., **α** = 0.1). We applied a relatively loose threshold for **α** without multiple test compensation in order to be more inclusive in the initial screening stage.

### Comorbidity association

There are 832 main discharge diagnoses after collapsing the ICD-9 codes to three digits. We construct the comorbidity association in the same manner as the medication variables are selected by removing diagnoses whose frequency is less than 10. Retained conditions are qualified by **χ** ^2^ statistic greater than 20 (i.e., significance level **α** = 0.001), resulting in 61 comorbid conditions highly associated with PUs.

### Bayesian networks

We conducted experiments to compare the performance of four search algorithms: greedy hill climbing, repeated hill climbing, Tabu search, and simulated annealing; two scoring functions: BDeu and MDL; two structure initializations: empty network and naïve Bayes; and five sets of features: Braden (B), medication (M), diagnosis (D), Braden & diagnosis (BD), and Braden & medication & diagnosis (BMD). All features except Braden are binary. Sensitivity (SENS), Specificity (SPEC), and Area Under the Curve (AUC) are used as metrics for purposes of comparison. We performed 100 trials of each experiment and report the average and standard deviation of SENS, SPEC, and AUC. In terms of the search algorithms, Tabu search outperforms most of results from greedy hill climbing, repeated hill climbing, and simulated annealing. The running time of repeated hill climbing is often long and yields poor results. Among scoring functions, BDeu performs better than MDL in hill climbing and repeated hill climbing algorithms, but both scoring functions are comparable in performance when used with Tabu search. In general, BDeu is very sensitive to the equivalent sample size parameter, and selecting an appropriate value can be challenging [29, 33]. We found that BDeu with a larger equivalent sample size parameter performs better than with the smaller one. Regardless of the search algorithms and scoring functions, using naïve Bayes to create the initial outperforms the performance of the Bayesian networks initialized with empty networks. In terms of feature sets, Braden & diagnosis gives the best average AUC, which is consistent with our previous study [6].

**Fig. 2:**
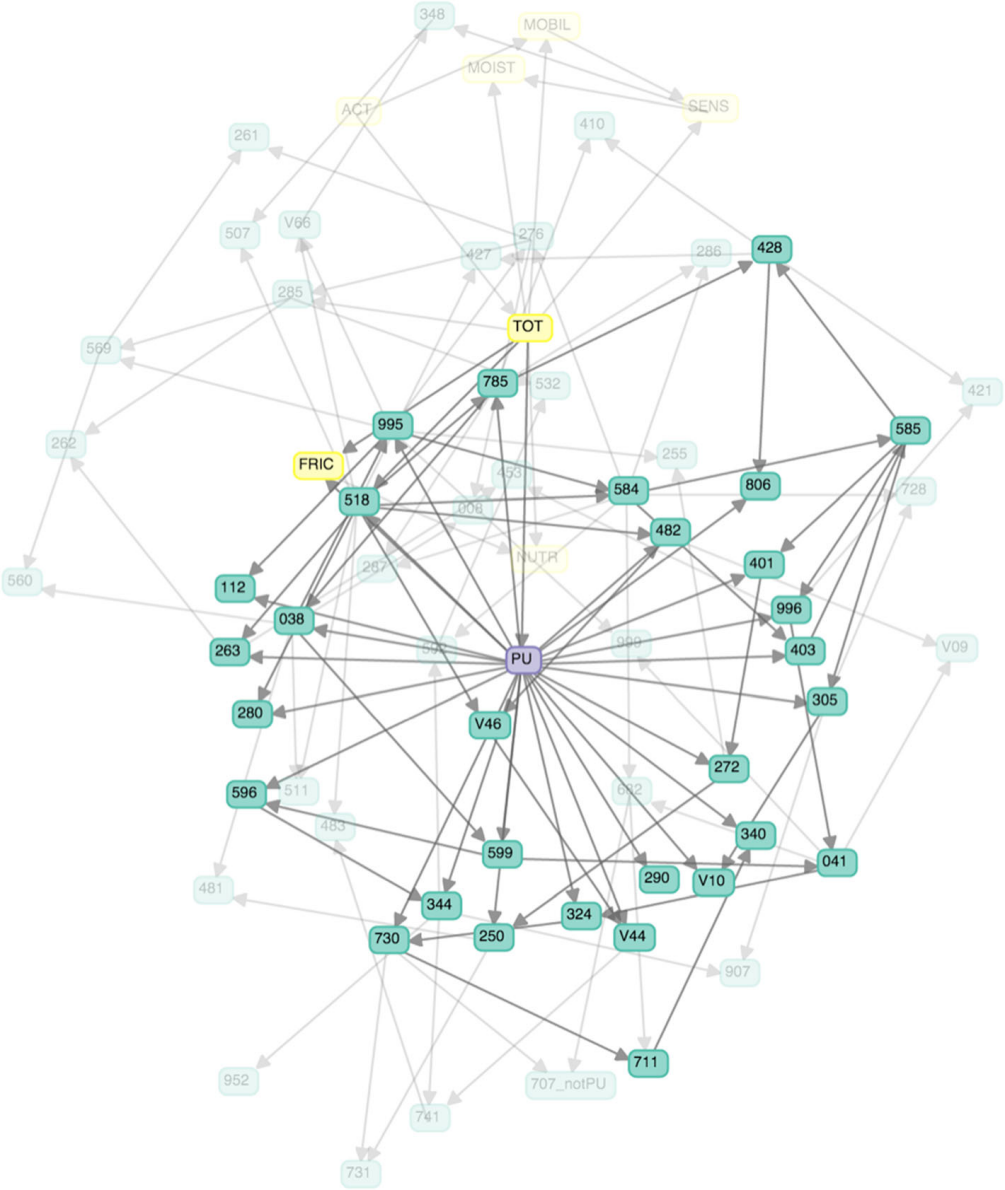
Bayesian networks for ICU data – The network with the best AUC is shown here. Highlighted nodes are in the Markov blanket of the node representing PU

Figure 2 shows a representative Bayesian network of Braden & diagnosis feature set from the 100 trials. The learned network consists of 69 nodes, each of which corresponds to a Braden subscale or diagnosis feature. The middle node labeled “PU” is a pressure ulcer diagnosis. The 32 highlighted nodes are the Markov blanket of PU (see Section Methods). In the Markov blanket of PU, there are 2 Braden subscales: total score and friction & shear. The other nodes represent diagnoses in form of ICD9 codes.

### Evaluation

A natural way to measure Bayesian network performance is to predict unobserved data. The data were randomly split into 2 sets: training (67%) and validating (33%) sets for 100 trials. The Bayesian network structure is learned from each training dataset. Sensitivity (SENS), Specificity (SPEC), Positive Predictive Value (PPV), Negative Predictive Value (NPV), and Area Under the Curve (AUC) are computed for each trial. Table 2 shows average predictive performance of each measure (row), where for each feature set (column) we use the best performing (as measured by AUC) combination of search algorithm, scoring function, and structure initialization, as reported below. The M feature set gives the lowest average AUC, 0.619 i.e., lower than the baseline (B). The D and BMD feature sets give fairly good average AUC, 0.810 and 0.819, respectively. The highest average AUC, 0.827, is from the BD feature set.

**Table 2:** Performance measures: mean (standard deviation) of Bayesian networks in five different feature sets: Braden (B), Medication (M), Diagnosis (D), Braden & Diagnosis (BD), and Braden & Medication & Diagnosis (BMD)

	B^a^	M^a^	D^a^	BD^b^	BMD^b^
SENS	0.021 (0.034)	0.002 (0.003)	0.315 (0.027)	0.455 (0.034)	0.478 (0.025)
SPEC	0.996 (0.006)	0.999 (0.001)	0.939 (0.005)	0.908 (0.006)	0.895 (0.007)
PPV	0.146 (0.201)	0.238 (0.385)	0.301 (0.022)	0.292 (0.184)	0.274 (0.015)
NPV	0.924 (0.002)	0.923 (0.001)	0.943 (0.002)	0.953 (0.003)	0.954 (0.002)
AUC	0.731 (0.018)	0.619 (0.016)	0.810 (0.012)	0.827 (0.011)	0.819 (0.011)

^a^MDL scoring function, Tabu search and naïve Bayes prior structure

^b^MDL/BDeu scoring functions give the same result, Tabu search, and naïve Bayes prior structure

We observed similar results in our previous study [6] in which the same sets of performance measures and features were evaluated on six different machine learning algorithms: linear regression, naïve Bayes, decision tree, *k*-nearest neighbor, random forest, and support vector machine.

We next study how our Bayesian network modeling performs against logistic regression (LR). We compare to LR because in our previous work [6], LR performed best among six machine learning models including SVM and random forest, and is simpler to tune. Three performance measures are used: sensitivity, specificity, and AUC. We compare 3 experiments: 1) a baseline method (“Braden”) which, mimicking a standard clinical assessment tool, thresholds a single Braden total subscale at 13, which maximizes AUC [5]; 2) Logistic regression (“LR”) on BD features; and 3) Bayesian network (“BN”) on BD features. We use BD features for the latter two because it gave the best AUC in our earlier performance study.

**Fig. 3:**
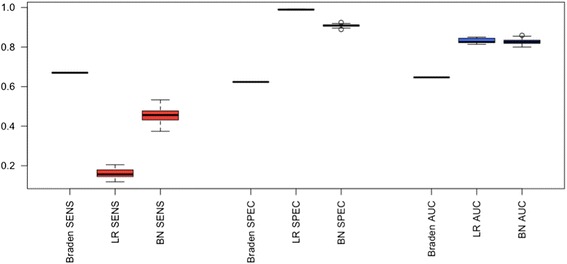
Box plot of SENS, SPEC, and AUC among Braden scale, logistic regression (LR), and Bayesian network (BN)

Figure 3 shows a box plot of our performance measures for these 3 experiments. The Braden baseline gives balanced, almost equal, sensitivity and specificity (0.670 and 0.623, respectively). While it maximizes AUC, it does so based solely on the single Braden total subscale feature. As a result, its AUC (0.647) is much lower than the other two, more complex models, demonstrating the limitations of using only one feature. For the other two experiments, in terms of AUC our Bayesian network model gives comparable performance to logistic regression (0.827 vs. 0.830). While Bayesian network has a slightly lower specificity (0.908 vs. 0.990), it also has almost three times the sensitivity (0.455 vs. 0.160). In other words, our Bayesian network model nearly triples the sensitivity at the cost of a slightly lower – although still high in absolute terms - specificity, while overall performance (AUC) remains comparable. We find this result promising because treating PU incidence is very costly while preventive measures are not. From the perspective of cost and patient quality of life, it is important to have high sensitivity to predict the patients who may develop a PU.

**Fig. 4:**
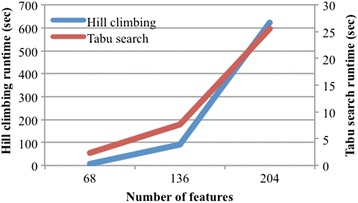
Scalability: Number of features

**Fig. 5:**
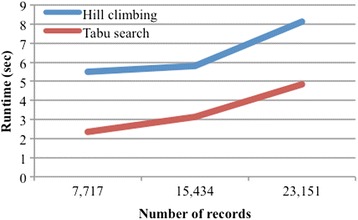
Scalability: Number of records

Lastly, we evaluate the scalability of Bayesian network learning, in terms of both number of features and number of records (patients). We took the best performing feature set, Braden and diagnosis (BD features) and created a larger 136 feature dataset from it by duplicating each of BD’s 68 features, and an even larger 204 feature dataset by triplicating BD’s features. Figure 4 shows the runtime of Bayesian network learning using greedy hill climbing and Tabu search algorithms. The runtime increases more significantly in larger number of features, which reinforces the importance of feature selection in our framework. To evaluate the scalability in terms of number of records, starting with BD, we created a 15,434 record dataset by duplicating each patient in BD, and an even larger 23,151 record dataset by triplicating each patient in BD. Figure 5 shows that the runtime for both greedy hill climbing and Tabu search increases only slightly more than linearly with the number of records. This suggests that Bayesian network learning can handle datasets with a moderate to large number of patients.

## Discussion

Four years of ICU EHR data are extracted from the information warehouse. We focus on patients who contracted PUs during their first ICU stay. Specifically, we select only the first hospitalization and filter out later hospitalizations to make sure that there is no impact from the previous PU incidence on the current hospitalization. Medications, diagnoses, and Braden features are used to develop predictive models, and univariate statistical analyses are carried out to reduce the number of model variables. A Bayesian network modeling approach is employed and we evaluate combinations of different scoring functions, search algorithms, and structure initialization methods for Bayesian networks. Compared to the best of a number of classical machine learning algorithms, the resulting Bayesian network model nearly triples model sensitivity at only a slight cost to specificity, while overall performance (AUC) remains high. High sensitivity is important in positively predicting patients who may develop a PU, as early intervention can be far less costly than treatment. The Bayesian network approach also provides an interpretable structure, allowing clinicians to understand, discuss, validate, and explain the logic behind the model. We select the risk factors highly associated with PUs using the Markov blanket property of the PU node. This approach reduces the number of PU risk factors and provides a more concise set of strong factors to clinicians for validation.

Our clinician collaborators identified many of the relationships depicted in Fig. 2 consistent with domain knowledge and clinical observations. For instance, it was obvious to them that ICD-9 main code 250 (Diabetes mellitus) is strongly related to PU incidence due to vascular circulation issues and (frequent) obesity associated with Diabetes. They also confirmed that many of the patients afflicted with disease code ICD-9 main code 806 (spinal cord injury) are very likely to contract PUs. The ICD-9 main code 995 (adverse effects) appearing as a child node of PU required more thought on their part. They realized that 995 appears as a child of PU because most PU patients in our data suffer from specific adverse effects of Sepsis (995.91) and Systemic Inflammatory Response Syndrome (SIRS, 995.9). In other words, non-obvious relationships in the data are gleaned from the Bayesian network.

Figure 2 can also be used to help confirm suspected associations. For instance, several studies show that PUs in diabetic patients often occur on extremities [34, 35]. Figure 2 shows an edge from PU to 250 (Diabetes mellitus). Other Fig. 2 associations include between PU and maladies of kidney (codes 403, 584, and 585), and cardiovascular issues (codes 428 and 785). Once again, both observations are consistent with findings from previous studies [28, 36]. Septicemia or sepsis (995 and 038) and respiratory failure (528 and 482) have been identified earlier as risk factors [37]. Further, we confirm that spinal cord injury (806), infection of the bone (324 and 730), and dementias (290) are also some of the risk factors of PUs as identified elsewhere [38–40].

Of the six Braden subscales, Braden total score and friction & shear appearing in the Markov blanket of the PU node indicate that they are the two most significant. Other Braden subscales i.e., activity, nutrition, mobility, sensory perception, and moisture are not as useful since they are more likely to have similar values for most ICU patients. ICU patients are likely to be sedate and in bed rest. Unsurprisingly, most diagnoses highly associated with PU incidence involve patients’ immobility including paralytic syndromes, spinal cord injuries, machine or device dependence, infections including sepsis and urinary tract infection, or imperceptions including dementias. In short, clinicians can benefit from the predictive model which helps them better understand the risk factors, leading to allocation of preventive measures and evidence-based risk assessment.

There are limitations pertaining to our study. The data are from a single institution; thus, interpretation of the finding is limited. The predictive models are constructed based solely on the data from EHRs and our data do not contain APACHE-II score, a severity of disease classification system. Consequently, we are unable to adjust for severity of illness, nor is severity of illness used as a predictor or PUs. The predictive models do not consider longitudinal analysis since temporal modeling would significantly complicate both the learning process and interpretation. We did not directly evaluate the robustness of learned Bayesian networks, for instance using different network similarity measures, parameter combinations, and perturbation models. As each of these variations deserves careful attention, we leave a comprehensive evaluation of the robustness of learned Bayesian networks as future work. Finally, the predictive power and risk factors of pressure ulcer incidence in this study are only based upon Braden scale, discharge diagnoses, and medication data.

## Conclusions

In this work we develop predictive models to help clinicians improve patient care. Motivated to assist clinicians, necessitating the use of an intuitive and interpretable model, we select Bayesian networks to serve our purpose. We present a three-step framework for predictive analysis of patient clinical data, consisting of data preprocessing, feature selection, and model construction. We apply our framework specifically to pressure ulcers in ICU settings, where we consider 86 diagnosis, medication, and Braden scale features extracted from a dataset of 7,717 patient EHRs.

We evaluate all combinations of Bayesian network models from four search algorithms, two prior structure initializations, two scoring functions, and five sets of features. Our model gives comparable overall performance to the best of classical machine learning algorithms, while nearly tripling sensitivity at only a slight cost to specificity with no sacrifice on high overall accuracy. We consider this promising since high sensitivity can better facilitate preventive care in patients likely to contract PU, which is less costly than treatment. From a qualitative standpoint, our clinician collaborators identified strong relationships between risk factors widely recognized as associated with pressure ulcers. These include cardiovascular, kidney, lung, spinal cord, bladder, bone issues or infections, dementias, diabetes, malnutrition, sepsis, friction & shear, and Braden total score. Identifying accurate risk factors of PUs is a key to comprehend disease burden and to improve pressure ulcer care. Our clinical collaborators found the Bayesian model useful in identifying dependencies between pressure ulcers and risk factors consistent with their own experience.

## Abbreviations

ADT^©^: Administrative System
AIC: Akaike’s Information Criterion
AUC: Area Under the Curve (Receiver operating characteristic)
B: Braden feature
BD: Braden & diagnosis feature set
BDeu: Bayesian Dirichlet with score equivalence and uniform priors
BIC: Bayesian Information Criterion
BMD: Braden & Medication & Diagnosis feature set
BN: Bayesian Network
CPOE: Computerized Provider Order Entry
D: Diagnosis feature set
EHRs: Electronic Health Records
FET: Fisher’s Exact Test
fNML: factorized Normalized Maximum Likelihood
ICU: Intensive Care Unit
IW: Information Warehouse
LR: Logistic Regression
M: Medication feature set
MDL: Minimum Description Length
NPV: Negative Positive Value
OSUWMC: The Ohio State University Wexner Medical Center
PPV: Predictive Positive Value
PU: Pressure Ulcer
SD: Standard Deviation
SENS: Sensitivity
SNP: Single Nucleotide Polymorphism
SPEC: Specificity
